# A short natural history of mental time travels: a journey still travelled?

**DOI:** 10.1098/rstb.2023.0402

**Published:** 2024-09-16

**Authors:** Mathias Osvath, Mikael Johansson

**Affiliations:** ^1^ Cognitive Science, Lund University, Lund 221 00, Sweden; ^2^ Department of Psychology, Lund University, Lund 221 00, Sweden

**Keywords:** mental time travel, cognitive evolution, deep time cognition, episodic memory

## Abstract

Tulving’s introduction of episodic memory and the metaphor of mental time travel has immensely enriched our understanding of human cognition. However, his focus on human psychology, with limited consideration of evolutionary perspectives, led to the entrenched notion that mental time travel is uniquely human. We contend that adopting a phylogenetic perspective offers a deeper insight into cognition, revealing it as a continuous evolutionary process. Adherence to the uniqueness of pre-defined psychological concepts obstructs a more complete understanding. We offer a concise natural history to elucidate how events that occurred hundreds of millions of years ago have been pivotal for our ability to mentally time travel. We discuss how the human brain, utilizing parts with ancient origins in a networked manner, enables mental time travel. This underscores that episodic memories and mental time travel are not isolated mental constructs but integral to our perception and representation of the world. We conclude by examining recent evidence of neuroanatomical correlates found only in great apes, which show great variability, indicating the ongoing evolution of mental time travel in humans.

This article is part of the theme issue ‘Elements of episodic memory: lessons from 40 years of research’.

## 1. Introduction

When Endel Tulving detailed the concept of ‘episodic memory’ in his book *Elements of episodic memory* [1], he identified a central feature of our cognition that would forever change memory research and profoundly impact other areas of the cognitive sciences.

In the book’s opening paragraph, Tulving used the metaphor of mentally travelling in time to describe how episodic memories qualitatively differ from other types of memories. He also declared that episodic memories and the mental travels they enable are uniquely human. He claimed that no other species has quite the same ability to re-experience happenings from the past and know that these experiences refer to an event that occurred at another time and another place. Reflecting on these statements two decades later, Tulving revealed that they were crafted when behaviourism was still restraining memory research and aimed to emphasize that human memory and learning encompass dimensions beyond non-human animals [2].

In Tulving’s well motivated quest to point out that there is more to human cognition than mere stimulus–response patterns, he, in effect, implied that the cognition of all other animals is restricted to such relationships. Perhaps this was not his actual viewpoint, but his interest lay in humans and not elsewhere. Even though his new memory concept opened avenues for understanding human cognition, its definition was not equally beneficial for research into cognitive evolution. A strong dichotomy arose. As animals, per Tulving’s definition, lack an episodic system, they are ‘stuck in time’. This argument has come in different flavours and theoretical guises (e.g. [2–4]). Proponents of such ideas predominantly have a human-centric interest, mirroring Tulving’s orientation, and lack a deep time perspective on the evolution of cognition. They may express evolutionary considerations but limit these to the recent hominin lineage and remain doubtful that the skills exist even in the other extant great apes.

Focusing on human cognition and psychology is an important pursuit, but it is too limited to explain non-human cognition or cognitive evolution. Moreover, to understand why humans have the ability to mentally travel, one must consider the natural history of cognition. Existing research on corvids and apes has already questioned whether mental time travel, especially into the future, is unique to humans (e.g. [5–12]). However, we think there is a lack of a grander perspective that asks why we can mentally travel and why some other species, even those as phylogenetically distant as birds, show similar abilities. Considering such perspectives can be fruitful even if one’s primary interest is in humans. We agree with Cisek [13] that cognitive theories based on a phylogenetic approach, where each cognitive function is seen as an elaboration or extension of a preceding one, provide a fuller understanding of cognition than studying humans alone. These approaches also highlight how large portions of the brain are integrated into what we perceive as distinct cognitive concepts, like mental time travel.

This paper provides a brief natural history of mental time travel to illustrate how cognitive evolution relies on releasing constraints. It focuses on the key events in deep time. To become a mental time traveller, one must first become a traveller and possess the biological need and ability to embark on physical travels. Events occurring hundreds of millions of years ago, seemingly unrelated to our current capacities, were indeed crucial. We start with fish, continue with the vertebrate invasion of land and end with the advent of endothermy. Thereafter, we turn to explore how much of the human brain is involved in what we call mental time travel, emphasizing its integral role in our perception and representation of the world rather than as a distinct memory system feature. This reveals how much previously evolved mechanisms are involved in what is often perceived as a newly arisen cognitive mechanism. Finally, drawing from recent discoveries in the brains of great apes, we argue that we can observe the ongoing evolution of mental time travel, seemingly encompassing abilities that Tulving identified as pivotal for episodic memories.

## 2. Fish

The first brains were selected for enabling navigation. A mobile organism composed of numerous specialized interacting cells requires coordination of its steering, and that is what a brain provides [14]. Hence, the brains’ original task was tuned to travelling, but there was still a long way before they afforded mental travelling. Some hundred million years after the origins of brains in nematode-like creatures, fish took some crucial steps in the direction of mental travel.

While fish were the first to venture onto land with bony feet, we do not categorize tetrapods as fish. Thus, fish are paraphyletic. However, the tetrapods’ direct ancestors are lobed-finned fish; these fish are more closely related to us than to sharks, for example. Consequently, critical brain adaptations in fish underpin our cognitive framework. Fish were the first to evolve a mental model of the surrounding world and a model of themselves, two central aspects of mental time travel.

For these early vertebrates, navigation became increasingly important, so a budding hippocampus was formed. This enabled far more flexible travelling behaviours than simple conditioned reflexes allow. Memories of sights and smells, their spatial arrangement and their sequence in the environment collectively formed a mental map of the outer world [15]. Teleost fish use such allocentric spatial maps and can reach goals regardless of starting positions [16]. They may also use shortcuts and novel routes. Essentially, they rely on integrated scenes rather than individual cues for navigation. Lesions to their hippocampal homologue significantly impair these abilities, underscoring the shared origin of the hippocampus and mental maps.

Another remarkable innovation is the cerebellum [17]. One of its fundamental roles is distinguishing the self from the external world by top-down modelling of motor movements and their sensory outcomes. This allows the animal to differentiate whether it is causing a change in the world or if environmental factors are responsible for the change. For instance, when swimming towards a rocky obstacle, the animal recognizes its own movement bringing it closer, while realizing the rock remains firmly in place.

It is unknown whether these models are integrated into the fish brain, but it is clear that the first version of a mental map and a ‘self’ had marine origins. However, mental time travel needed air and land to continue its journey.

## 3. The invasion of land

Water has a remarkable impact on the range of vision, as even the clearest waters dramatically hinder the light waves compared with air. The fish brain is primarily adapted to this visual ecology, where objects appear out of nowhere suddenly and where the horizon lies a few swim strokes away [18]. Most things a fish can see are within striking range, and therefore they possess extremely quick escape responses, mediated by the so-called Mauthner pathway in their brains. Essentially, their perceptual worlds are limited, prioritizing reaction over contemplation.

However, once you gaze above the water’s surface, particularly over land, a transformation unfolds. The visual range increases at least a hundredfold. And, if you evolve a neck that enables head movement, over a million times more visual space becomes accessible. MacIver *et al*. [18] have argued that this fact pulled some fish onto land and eventually turned them into tetrapods. On land, fish gained extended vision, spotting unsuspecting prey. Insects had already colonized land some 50 million years earlier and were long since oblivious to being caught by vertebrates. In the early days, there was probably a food bonanza along the shores where intrepid fish had started their leaps into the air and onto land. But soon, prey likely adapted, and competition increased among the insect-munching fish, driving them further upon the land.

MacIver *et al*. [18,19] assert that terrestrial conditions, especially visual ecology, facilitated the evolution of planning skills in vertebrates. The terrestrial environment abounds with various types of substrates and objects that may or may not be locomoted upon. Thereby, the amount of possible processable information increases; hence, there is room for a range of brain adaptations. The further an animal can see, the more time it has to predict the future. When not confined to a few body lengths of vision, the possibility arose to take deliberate actions and plan sequences before encountering the distant prey, predator or food patch. The extremely quick reactions mediated by the Mauthner neurons would become counterproductive. In other words, terrestrial conditions incentivized mental time travel, particularly into the future.

However, it is improbable that such planning adaptations emerged immediately in the earliest tetrapods. Amphibians, representing the initial land-dwelling vertebrates, appear confined to a relatively limited world. Their brains still share several similarities with lobed-finned fish, and notably, they retain Mauthner neurons, which are absent in other land vertebrates. Their hippocampi are not markedly derived and lack several of the adaptations that came later [20]. Despite their capacity for allocentric mental mapping in navigation, they sometimes resort to simpler forms of learning [21]. Their cerebella are not majorly different from their predecessors. They are actually smaller than in lobed-finned fish [22], and they appear not to have evolved any other models of the ‘self’. Although amphibians are land vertebrates, they remain firmly tied to water; their early life stages occur in water, and they still require constant moisture to prevent dehydration. Their low-energy lifestyle is, to a large degree, governed by water temperature, as excessive basking leads to fatal dehydration. It seems as if amphibians do not need any significant additional brain adaptations for navigation compared with fish. Thus, to fully exploit the opportunities for mental time travel offered by land, one must break free from water’s constraints and explore the wider world. Physical and mental travel abilities are intertwined; there is no gain in evolving brain structures enabling elaborate mental travels if physical travels are constrained. However, a leap in the evolution of land travel occurred 325 million years ago.

## 4. The ectotherm amniotes

Approximately 60 million years following the invasion of land, a grand invention of nature scurried around on the forest floors. This was the stem amniote, the common ancestor of all reptiles, non-avian dinosaurs, birds and mammals. It could do what its amphibian predecessors could not. It produced eggs that hatched on land. It had thick keratinized skin that kept the moisture inside its body. It respired by expanding and constricting its rib cage. This creature no longer needed water for anything other than drinking. Amniotes moved out to dry areas and raised their activity levels, thanks to a well developed ability to bask and better gas exchange from dry air. They also adapted their locomotor anatomy, which increased the range of motion in their legs. Not surprisingly, their brains started to develop.

The mammal and the bird amniote forebears were ectothermic and likely rather similar initially. Today, extant amniotic ectotherms only exist in the sauropsid lineage. Lizards and the tuatara (*Sphenodon punctatus*) are arguably as close as we get to the early amniotes. Their hippocampi notably differ from those of amphibians. Unlike amphibians, their enlarged hippocampi are laminated and no longer receive direct sensory input from the olfactory bulb and thalamus. Reptiles’ hippocampi use sensory inputs already processed by their expanded, compared with amphibians, pallial telencephalon [20]. Precisely how these marked differences translate into behaviour is not well understood owing to there having been few comparative cognitive studies on amphibians and reptiles. Still, it appears clear that lizards rely on a much more extensive repertoire of spatial learning than amphibians [21,23]. Most likely, their memory capacity also increased; it has, for instance, been shown that the crevice spiny lizard (*Sceloporus poinsettii*) will return to a location where it found food 24 h earlier [24]. Such feats have yet to be reported for amphibians.

Considering the adaptations in the reptilian brain, one can ask if there is an emerging ‘self-concept’ beyond the split-second self-modeller of the cerebellum. A more developed model of a ‘self’ likely necessitates brain structures integrating information from various parts of the brain and having top-down functions akin to the mammalian prefrontal cortex. Interestingly, a small area in the crocodilian brain similar to the nidopallium caudolaterale (the prefrontal cortex analogue in birds) has been discovered [25]. However, given that crocodilians belong to the archosaurs—the same group as birds and other dinosaurs—which originated roughly 240 million years ago and underwent numerous new adaptations not observed in other reptiles, it is conceivable that this feature may be absent in other reptiles and hence not present in the stem amniote.

Despite the advancements in terrestrial and mental travelling abilities of ectothermic amniotes, they were constrained in their travelling capacities because they could not move continuously over large areas, and they were restricted by their behavioural thermoregulation.

## 5. The heat from within and the origins of gluttony

The evolution of endothermy remains debated in theoretical biology. However, its behavioural and cognitive consequences, apparent in both Synapsida and Sauropsida, are more tractable than the reasons for its evolution or timing. The shift to true endothermy ignited a neurocognitive revolution. Endotherms boast at least 20 times the neuron count of same-sized amniotic ectotherms, with many surpassing 75 times the expected neuron count of ectotherms [26]. This significant neural expansion represents a novel cost/benefit dynamic of neural tissue. The increase in the number of neurons, being energetically demanding, is compounded by the fact that endotherms also use 20 times more energy than ectotherms [27]. What appears to be a paradox is likely the opposite: the substantial increase in expensive neurons seems essential for procuring the additional energy required. In this context, one should also acknowledge that higher body temperatures benefit neuron efficiency [28,29]. Additionally, elevated temperatures reduce the energetic costs associated with action potentials [30].

In endotherms, a larger relative brain size is associated with a longer lifespan, whereas in reptiles, a relatively larger brain leads to a shortened life [31]. When ectotherms allocate resources to costly neural tissue, it competes with other vital bodily processes, leading to quicker deterioration. However, in endotherms, increased brain capacity appears to support these processes. Endothermic brains can also allocate energy to support the needs of others’ bodies, as observed in parental care, unlike ectothermic amniotes, which typically show limited engagement with offspring [32]. The greater the brain size of an endotherm, the more extensive care it tends to provide for its young. Broadly speaking, the endothermic brain appears to be a dedicated energy provider, whereas the ectotherm brain performs a more precarious tightrope act in balancing its benefits with the energy it consumes.

This shift in brains and cognition is likely closely tied to the novel approach to food in endotherms. Never in the history of animals has so much food been consumed by individual animals as with the endotherms. The 20-fold increase in energy is fuelled by edibles. In contrast, ectotherms rely almost exclusively on the environment for their body temperature and can endure long periods without eating. They require food only for maintenance, growth and reproduction [33]. Extant endotherms eat roughly 10 times as much food by weight as similarly sized active amniotic ectotherms [34]. Therefore, many of the new brain elaborations in endotherms appear geared towards more efficient foraging [35]. This efficiency is greatly facilitated by shedding several ectothermic constraints. Endotherms can cover vastly greater distances, much less affected by shifting weather conditions or night-time coolness. Now, the mind’s travels in time and space have become a useful expansion.

## 6. The endothermic brain hypothesis

Despite sharing a common ancestor in the stem amniote, as far back as 325 million years ago, the warm-bodied birds and mammals converged in their cognitive and brain functions [36]. Besides the aforementioned extreme expansion of neuronal numbers in both groups, they also independently evolved an isocortex, or rather mammals did, and birds (or some non-avian dinosaurs) evolved equivalent pallial functions. Additionally, they, respectively, evolved a prefrontal cortex and a nidopallium caudolaterale, which, as mentioned, might have had an ectothermic precursor, at least in the Sauropsida. Naturally, their hippocampi also majorly grew in number of neurons and became densely connected to the isocortex and the pallial areas.

The isocortex and its avian pallial counterpart facilitate the remarkably neuron-intensive ability of perceptual simulation [37]. These regions serve as the neural basis for generating a model of the perceived world, a crucial aspect of endothermic cognition. They simulate incoming information against past experiences, constantly filling in gaps in sensory input. However, these predictive simulations, rooted in the past, also play a vital role in mental time travel.

Integrating perceptual simulations with the pre-existing hippocampal mental map establishes a cognitive realm where actions and decisions can be assessed before encountering reality. In Popperian terms, this system lets hypotheses die in our stead. Edward Tolman observed behavioural evidence of this endothermic planning ability in the 1930s [38]. He noticed that rats often paused and glanced in different directions when they reached forks in mazes prior to deciding their next move. This behaviour, coined as vicarious-trial-and-error learning, indicated that learning occurred through mental simulation rather than direct experience. Tolman proposed that the rat mentally explores alternative options before making a decision. He was not the only one suggesting mental simulations in animals; for example, Wolfgang Köhler in Europe and Robert Yerkes in the USA studied primate behaviours in the 1920s, attributing them to elements of such simulations [39,40]. However, this occurred during a time when neuroscience was still in its infancy, and the behaviouristic paradigm held sway, so their interpretations of their findings were not entirely accepted.

We now know that when rats exhibit this behaviour, it is associated with hippocampal place cells firing, indicating that different routes are represented when the rat is hesitating [41]. It was also recently shown that pigeons dream—a clear sensorimotor simulation—and reveal remarkably similar brain activation patterns to mammals, activating, for example, sensory and associative sensory areas [42]. As one of the authors later pointed out, the specific brain activations seemed to reveal that the birds were dreaming of flying and avoiding collisions with objects [36]. These are just two instances among numerous findings affirming that endotherms do indeed simulate sensorimotor events. However, it remains uncertain whether any extant reptiles or certain species among them possess any precursors to these abilities.

Nonetheless, the crucial question remains: how do these abilities contribute to the efficient foraging essential for supporting the fuel-burning lifestyle of endotherms? The endothermic brain hypothesis contends that the novel neurocognitive elaborations mainly evolved to facilitate a certain type of foraging [35]. Foraging typically involves searching, at the core of which lies the exploitation versus exploration dilemma [43]. Whether to exploit known resources or explore for potentially better ones elsewhere is perhaps the most common quandary in nature. Since one cannot simultaneously do both, it often becomes a defining question, quite literally, for survival. Notably, the earliest brains and their hormonal systems were shaped by this existential question, a fundamental task that has persisted and been elaborated over time [43].

According to the endothermic brain hypothesis, the emergence of simulations, coupled with cognitive maps and other functions, facilitated what is known as model-based exploration, significantly enhancing the amount of energy that could be harvested. Model-free exploration is primarily random and lacks guidance towards any goal other than finding a resource, whereas model-based exploration relies on a belief-driven search using an environmental model [43]. With a memory-based map of the surroundings and their resources in relation to cues such as weather or time of day, and the ability to simulate various options before making a selection, an endotherm possesses precisely the tools needed to maximize its energy intake. It can leverage the promise of the future to optimize its energy acquisition.

Naturally, these abilities must vary among endotherms, and new functions and elaborations have certainly evolved across different taxa. Nevertheless, very few comparative studies across taxa have yet been conducted. We do, however, possess significant knowledge about ourselves and our closest living relatives. Tulving and many others have argued that mental time travel is a uniquely human skill. We, on the other hand, believe that human time-travelling abilities currently represent the most advanced skills in this regard, closely followed by our chimpanzee relatives and much likely by some bird species. However, there is still limited understanding of the latter. Therefore, it is time to end our travel through deep time, focus on ourselves and the other great apes, and acknowledge that these skills are likely still evolving.

## 7. Human mental time travellers

When introducing episodic memory, Tulving [44] contrasted it with semantic memory, comprising world knowledge, generic facts, concepts and their relations. Whereas episodic memory stores information about personal experiences in the spatial and temporal contexts in which they occurred, allowing us to relive those experiences later, semantic memory is not tied to personal experiences or specific events in time. Instead, it represents a more generalized repository of knowledge about the world and the language we use to describe it. Semantic and episodic memory were later conceived as two systems with distinct, or at least not entirely overlapping, neural underpinnings.

Although considered distinct systems, episodic and semantic memory are intimately intertwined. Consider, for example, how prior knowledge and past experiences influence our perception and comprehension of the world around us. The neural representation of an unfolding event is not merely dictated by the incoming sensory information but shaped by previous perceptual processing and applicable concepts, expertise, and records of past events, providing the basis for extracting meaning and generating predictions (e.g. [45]). The constructive nature of memory is evident during remembering when we reconstruct past personal experiences, again influenced by prior knowledge about the world [46–48]. Thus, semantic memory affects episodic memory. Conversely, episodic memories may form semantic memory over time as invariant information across accumulated experiences is abstracted into general knowledge [49]. These interactions shed light on the overlaps between episodic and semantic memory and the somewhat fuzzy boundary between perception and memory.

In Tulving’s conceptualization, the two memory types are associated with different levels of conscious awareness. Mental time travel that characterizes episodic remembering entails a first-person subjective re-experience of a past event, associated with ‘autonoetic awareness’, which is described as ‘the type of awareness experienced when one thinks back to a specific moment in one’s personal past and consciously recollects some prior episode or state as it was previously experienced’ [50]. Retrieval from semantic memory, on the other hand, involves just knowing something and is associated with ‘noetic awareness’ [51]. Thus, resonating with William James’ classic assertion, ‘Memory requires more than mere dating of a fact in the past. It must be dated in *my* past’ [52, p. 650]. The self-constitutes a fundamental part of episodic memory and the associated mental time travelling.

## 8. The human brain’s time machine

The human mental time traveller utilizes a broad network of brain regions that evolved over hundreds of millions of years. The continuous flow of incoming information is systematically represented and structured into a sequence of event models, each represented by a distributed pattern of brain activity across functionally specialized cortical regions devoted to processing various episodic features. Cognitive neuroscience models of memory hold that re-experiencing during remembering is based on the reinstatement of cortical processes that were active at the time of the original experience [53–57]. The reinstatement is made possible via cortical–hippocampal interplay. The hippocampus stores an event index during learning, pointing to the distributed cortical building blocks of an event. Retrieval occurs when a retrieval cue matches the stored hippocampal index, triggering pattern completion and the reactivation of the cortical traces and, thus, the subjective re-experience of the past event (for review, see [58,59]). This theoretical framework is supported by various kinds of data, including machine learning approaches to functional neuroimaging data that successfully decode the contents of retrieval based on classifiers trained during encoding (e.g. [60–62]).

The hippocampus interacts with two broad cortical networks [63] clearly present in primates [64]: the anterior-temporal (AT) and the posterior-medial (PM) networks (cf. the ‘what’ and ‘where’ streams [65]). The AT network includes the perirhinal cortex, amygdala, ventral temporopolar cortex and lateral orbitofrontal cortex and is considered to process content (specific entities such as faces and objects). The PM network includes the parahippocampal cortex, retrosplenial cortex, anterior thalamic nuclei, mammillary bodies, presubiculum, parasubiculum and components of the ‘default mode network’, including the posterior cingulate, precuneus, angular gyrus and ventromedial prefrontal cortex, and is held to process context and support recollection-based memories involving spatial and episodic context, scene perception, simulation of hypothetical events and aspects of spatial navigation and social cognition. Thus, while the AT network would be sufficient for recognizing individual items based on familiarity, the integrated memory representation is required for recollecting the item in context. The AT and PM processing streams converge in the hippocampus, highlighting the hippocampus’s critical role in episodic memory and explaining why selective hippocampal lesions impact recollection while sparing familiarity-based item recognition memory (see Yonelinas *et al*. [66] for a recent review). The model is also supported by functional neuroimaging data showing that the perirhinal cortex is sufficient for encoding memory for individual items, whereas the hippocampus and parahippocampal cortex build episodic memories with the item embedded in a situational context (e.g. [67]).

Episodic remembering is cue-dependent [1]. Retrieval cues in the present bring to mind past experiences by triggering the reactivation of stored memories, allowing us to revisit the past to inform current thinking and behaviour. The more significant the overlap between the processing engaged during encoding and retrieval, the greater the likelihood of successful retrieval. For example, we are more likely to remember information if we return to the physical setting or the emotional state in which the information was encoded [68]. One does not need to revisit the encoding context physically; the same benefit of an encoding–retrieval overlap has been demonstrated following mental reinstatement, i.e. simulation of the learning setting [69].

A longstanding view holds that eye movements are critically involved in episodic memory and that gaze behaviour may assemble and organize visuospatial relations across time and space into coherent memories [70,71]. Recent research confirms these ideas by demonstrating that the visual sampling behaviours observed during encoding are replayed during episodic remembering when we mentally reconstruct events and simulate experiences from our personal past [72].

Hippocampal contributions to cognition are not limited to memory. The hippocampus has long been associated with spatial navigation, already in fish as mentioned above, and it has been further demonstrated in animal work that hippocampal place cells fire in a location-specific manner [73]. This observation gave rise to the cognitive map theory, suggesting that a critical role of the hippocampus is to create and uphold spatial maps of the surroundings from an allocentric perspective [74]. Human data indicate that the hippocampus plays a crucial role in abilities including spatial navigation [75], mental imagery [76], counterfactual thinking [77] and simulating a plausible personal future event [78,79]. Given its central position as a convergence zone, the hippocampus may have evolved to form, maintain and reactivate cohesive event representations encompassing distributed activation patterns across multiple cortical areas. Data suggest that this is true for representations not only of direct experience of the world but also of simulated experience, thus providing a fundamental basis for mental time (and space) travelling.

Frontoparietal regions that form part of the default mode network are activated during successful remembering, and event-specific reinstatement has been observed in the angular gyrus [80]. Data are consistent with the idea that the left angular gyrus integrates multimodal event features [81–83], which is of great importance for the subjective experience of remembering. Furthermore, research suggests that the egocentric, first-person perspective so characteristic of episodic remembering is mediated by parietal regions, including the left angular gyrus and the more medial precuneus (cf. the ‘mind’s eye’) [81,84,85]. As proposed by Simons *et al*. [86], connectivity between the precuneus and angular gyrus might bind multimodal episodic details within an egocentric framework into the kind of first-person-perspective representation that underlies the subjective re-experiencing of past events.

## 9. The still-evolving human and ape mental time traveller

The neural regions described thus far support subjective first-person experiences of the world, driven by direct perception, mental imagery, reconstruction of the past, and future simulation [87]. They provide the basis for detaching from the present, allowing daydreaming and counterfactual thinking, rehearsing and reanalysing past experiences and planning for the future. These adaptive excursions into a different time and space change us as they form new memories and revise old ones, ultimately influencing how we navigate our future (e.g. identifying upcoming challenges and preparing ways to overcome them). However, there may be times when it is essential to keep track of the source of our memories—whether they stem from reality or imagination. As the surrealist filmmaker Luis Buñuel said, ‘A good memory is needed to keep the many lies straight’.

The prefrontal cortex houses a range of cognitive control mechanisms that interact with memory. The medial anterior prefrontal cortex appears engaged in reality monitoring [88,89], the post-retrieval process of discriminating between memories acquired through perception of external reality and memories generated internally by imagination [90]. A vast amount of research has highlighted how reality-monitoring failures can range from benign confusions between the perceived and the imagined to more malign characteristics of mental illness, such as hallucinations observed in schizophrenia (see Simons *et al*. [91] for a review). Interestingly, research has shown a remarkable inter-individual variability in reality-monitoring performance (also in the healthy population) related to variations in functional activity and structural morphology in the medial anterior prefrontal cortex. In particular, the paracingulate sulcus with late sulcal ontogenesis shows high inter-individual and inter-hemispheric variability and is present in approximately 80% of individuals in at least one hemisphere [92]. Buda *et al*. [93] demonstrated that bilateral absence of the paracingulate sulci in a healthy population was associated with impaired reality monitoring and metacognitive performance compared with participants with a paracingulate sulcus in at least one hemisphere (see Simons *et al*. [86] for a recent review). Although these findings require further empirical validation, the available data suggest that a prominent aspect of mental time travel, central to Tulving’s definition—the self-related quality of episodic memories, being able to distinguish between experiences resulting from actual external events and those from simulation—is not ubiquitous in human cognition.

Recent work on sulcal development in primates suggests that the paracingulate sulcus is a brain innovation specific to the Hominoidea. Although previously considered a unique feature of the human brain, the paracingulate sulcus is actually present in 36% of chimpanzee brains, 39% of bonobo brains, 23% of gorilla brains and 50% of orangutan brains, but not in the brains of baboons, macaque, gibbons or siamangs [92,94]. Analysis of cytoarchitectonic and resting-state functional magnetic resonance imaging data further indicates that the chimpanzee paracingulate sulcus is homologous to the human paracingulate sulcus [95]. These findings, combined with the presence of a human equivalent default mode network in chimpanzees [96] but not in non-hominoid primates [97], suggest that fundamental components of mental time travel are not unique to human cognition.

Indeed, these statistics indicate the ongoing evolution of mental time travel skills in both humans and other great apes, tracing back at least to our common ancestor approximately 15 million years ago. The essential self-monitoring aspect of Tulving’s definition of episodic memories does not seem exclusive to humans, at least when considering brain correlates. Future work should aim to assess reality-monitoring functions in non-human great apes. The perspective proposed here—that the paracingulate sulcus and episodic memory, traditionally considered uniquely human traits, might be shared by all great apes—is a hypothesis that requires further empirical validation.

The selective pressure for reality-monitoring does not seem extremely strong, given its relatively slow increase over millions of years. Whether memories originate from external reality or past mental imagery and simulations may not always matter. In most cases, the utility of memory lies in its ability to inform decisions and actions, regardless of its source. Thus, both real experiences and imagined scenarios can contribute to effective problem-solving and future planning. The precise advantages conferred by this ability remain to be specified. Speculatively, it could be argued that memory-based predictions are associated with less certainty when founded on past simulations or experiences from others compared with direct experiences of reality. Therefore, as the capacity for mental simulations increased in the evolving brain, leaving records of experiences with varying predictive value, so did the need to monitor their source. Related speculation concerns the consequences of language development and the exposure to the actual or simulated experiences of others, necessitating a greater need to keep track of the original source. We assert that the question of why reality monitoring evolved is empirically tractable through the study of both humans and other Hominidae family members.

In our view, it is highly probable that apes possess episodic memories and can utilize them effectively in mental time travels. The neuroanatomical and neurofunctional discoveries show striking similarities to humans in areas crucial for our mental journeys. This, combined with abundant behavioural evidence on their memory systems [98]—including advanced selective memory-retrieval functions [99]—and planning skills (e.g. [5–8,100,101]) not observed in monkeys or young children [102–104], makes alternative explanations less plausible. Differences from humans may stem from differences in computational capacity due to fewer neurons and likely from the absence of language in apes, which significantly impacts cognitive abilities related to mental time travel. Mental time travel has been suggested as a prerequisite for the evolution of symbolic language [105].

## 10. Concluding remarks

The history of mental time travel is the story of the mind’s extension into space and time, mirroring key events in deep time, increasing the body’s ability to venture further into the environment. The human capacity for mental time travel depends heavily on our inner fish brain and the gluttony sparked by endothermy. When we engage in mental time travels, intricate interactions occur among an array of brain regions, some of ancient origins. Mental time travel cannot be viewed as a distinct and dedicated skill but as an outcome of our evolved perceptual and memory systems—so intertwined that their labels become metaphors rather than real cognitive entities. As pointed out by others (e.g. [13]), psychological terms and concepts are not as clear cut as they appear and even risk leading us astray when trying to understand the true workings of cognition. To fully understand our cognitive skills of today, we need to consider their deep phylogeny. Evolution can only work on what is present, and new abilities result from re-moulding previous ones. Despite this, modern psychological terminology often resembles engineering jargon, describing specialized system components designed for one specific purpose.

Indeed, the human mental time-travelling network exhibits uniqueness in several aspects of its parts and functions, as far as we know today. However, it begs the question: is it beneficial to characterize mental time travel as exclusively human? To answer this, one must pinpoint precisely which components of the network turn the process into true mental time travelling. As we have outlined, a vital element of this system, as defined by Tulving, is the awareness that a remembered event happened to oneself rather than being an imagined event or an experience told by someone else. This ability shows a great deal of variability among healthy individuals and has neural underpinnings that we share with the other great apes. Should we thus consider omitting this element from the definition? Such deliberations do not seem very enlightening regarding understanding the nature of cognition. Instead, we suggest acknowledging that mental time travel may exist along a continuum, particularly in endotherms, encompassing diverse forms of self-representation and simulation abilities. Embracing this notion, already bolstered by empirical evidence from brain studies and behaviour, would allow research to explore variations among different taxa and their underlying reasons, ultimately enhancing the comprehension of our own cognitive abilities.

## Data Availability

This article has no additional data.
